# Glass Eyes: Their Manufacture and Cost

**Published:** 1887-05-21

**Authors:** 


### Glass Eyes: their Manufacture and Cost.

By One who Has to Use Them.

Happily, it is not a matter in which many of us are immediately concerned; nevertheless it is interesting to learn that, like most other items of personal expense, the cost of an artificial eye has been steadily tending downwards of late. These curious articles used to be made almost exclusively in Paris, where it was said at one time that from twenty to twenty-five thousand a year were turned out. It will be easily understood that a life-like imitation of the human eye is a matter for an artist, and twenty years ago the Parisians were the only people who had brought artistic skill to bear on this odd manufacture—odd in a very literal sense, by the way, for only occasionally does it happen that artificial eyes are made in pairs, though it does now and then occur. London could make eyes for dolls or for stuffed animals, but anyone who required a really well-executed eye for his own head, had to go to the Faubourg St. Honoré.

That is no longer so. There are manufacturers in London who can very fairly compete with the best of continental makers, and the downward tendency of prices is said to be attributable to the large number of artificial eyes imported from Germany. There appear, indeed, to be several parts of Europe in which this queer business is now carried on quite as successfully as at Paris, and competition has become a good deal keener than it formerly was. A good eye is still a somewhat expensive luxury, and, like all works of art, will always command a good price; but those who are content with a second-rate article may get it a good deal cheaper than they could at one time.

These things are not always to be regarded as "luxuries"—things, that is to say, not really necessary, and adopted merely for the sake of appearance. There are many persons who find it absolutely imperative upon them to conceal a defect which nature or accident may have inflicted upon them. So severe is the competition for employment in these competitive times that a man or woman with an obviously defective eye will stand no chance at all in many spheres of activity, even though they may be more competent with one eye than many others with two. When, however, it is stated that an artificial eye costs from fifteen shillings to a couple of guineas, and that it will last not more than a twelvemonth, it will be seen that this necessity for presenting a good look-out to the world is one that many persons may find somewhat burdensome.

The artificial optic, though commonly spoken of as a "glass eye," is really made of a species of enamel, which, though seemingly as hard as the hardest glass, is gradually eaten into by the corrosive action of the tear-secretion. Tears, according to Professor Faraday, are merely chloride of sodium, carbonate of lime, and general folly; but so pungent and active is the tear-fluid in its nature, that Mr. Brudenell Carter, in his popular work on eyesight, tells us that when, as sometimes happens, the tear-passages are closed, and there is an overflow of moisture down the cheek, a most disfiguring open channel will in course of time be worn away. This corrosive fluid eats away the surface of the enamel, rendering it rough, and giving it the appearance of a dead eye. Messrs. Gray and Halford, of the Goswell Road, who are among the best manufacturers in London, tell us that the smoothness and lustre of the artificial eye may be restored to some extent when the corrosion is not very far gone, but, in a general way, about twelve months is the utmost length of time for wearing even the best of enamelled eyes.

Some few years ago it was said that one of the Forest Cantons of Switzerland—Uri—was turning out these embellishments of the human face in materials that were capable of withstanding the chemical activity of the tear. They had silicates and other mineral substances in their neighbourhood which enabled them to excel all other makers in the durability of their eyes. What degree of truth there may have been in this we are not prepared to say. Of course no London manufacturer will admit for a moment that their wares can be excelled by the Swiss or the Parisians, or by anybody else; and certainly it is difficult to conceive of anything much closer in its imitation of the natural orb than the workmanship of some of the best of London makers. By the way, it may not be generally known by those who, fortunately, have never had occasion to inquire into the mysteries of this subject, that though the natural organ of sight is really an orb, a true sphere, or nearly so, a little less than an inch in diameter in the adult, the artificial eye is not commonly used in this form. It is merely a delicate shell of enamel, representing the front of the eyeball. When the eyeball remains, the enamel shell is deftly fitted over the front of it; if not, it is adjusted within the eyelids, as though the eyeball were actually there. There are six muscles concerned in the movements of the optic globe. Of course, it sometimes happens that by disease or accident these muscles are destroyed or are disabled. In that case, it need hardly be said there can be no natural movement of the artificial eye. But when the muscles remain unimpaired, the enamel presentment of the eye-front, whether the globe be behind it or not, will move in correspondence with its living fellow, the eye on the other side the face.

If the artist has done his work skilfully, the disfigurement of the face will be entirely repaired, even more effectually, perhaps, than would be the case if complete success should ever attend the very extraordinary experiments reported on a year or two back at the Séance de Chirurgie of Paris. Five successive attempts were made to transplant the eyes of animals to the heads of human patients, and one of them was said at the time to have proved successful. In this case the eye of a rabbit was transferred to the head of a patient thirty-five years old. There was no expectation at the time the experiment was undertaken that the transferred eye would enable the patient to see, but, according to the scientific report, the transference was so signally successful, that it seemed not altogether improbable that the strands of nerve-tissue remaining in the human head would grow into the engrafted eye. At the time of the report it was deemed too early to permit of anything very positive being expressed as to the chances of this, and we have since heard no more of it, and it may be presumed that the expectation of any such union was not realised.

The great majority of eyes required in Europe are grey, but, of course, the artist in these wares has to lay himself out for a good many colours, and that not only in the iris but in what is commonly spoken of as the white of the eye. This may be anything but white, and indeed, strictly speaking, can rarely or never be imitated by anything near a pure white, any more than the pearliest of teeth could be. The colour of the "white" of the eye may range through innumerable shades, from something approaching a pure white to a dark blue or a bilious yellow very much bloodshot. All the peculiarities of the living eye have to be carefully imitated if a real success is to be attained, and it not unfrequently happens that fastidious customers are dissatisfied with the match, and return the eye to the maker as they would do a misfitting coat or a bad likeness. These articles go into stock, and customers to whom price is of greater importance than great nicety of imitation, sometimes get these at a reduction, or they are exported to other lands where people are not so particular. It is said that some of the inferior kinds of enamelled eyes are disposed of for bodies that are embalmed, and for which any passable presentment of the human eye is found to suffice.

Of export orders for glass eyes a good story is told by an anonymous writer, who says that a Haytian general sent his commands to a European manufacturer, who exerted all his skill, hoping to bring in business by a successful re-illumination of so distinguished a face, if not a reward in the shape of a foreign decoration. To his great surprise, however, the general returned the masterpiece. It had, he said a tinge of yellow in it, and this unpleasantly reminded him of the Spanish flag. He was determined to have no Spanish colours in his eye. He would have only the colours of his own country, and the new eye had to be made for the great man in the Haytain colours of green and red.

People who can afford to be particular in these matters will sometimes give a good deal of trouble in getting a good match, or what satisfies them as such, but the match having once been secured, an arrangement is sometimes made by which a maker undertakes to keep up a regular supply for an annual payment, just as paterfamilias will sometimes pay the family doctor an annual stipend to keep the family duly in medicine. These are the plutocrats among glass-eye wearers, and it is not an unusual thing for customers of this class to keep by them two or three eyes to be worn at different times in the day. Of course, everybody knows that the less the light the larger becomes the pupil of the eye. When light is strong the grey or brown or blue curtains of the iris are drawn to, and, obviously enough, if one eye were thus amenable to the influence of varying light while the other were to remain unchangeable, it would thwart all the care and skill of the artist and make the artificiality of one eye unpleasantly apparent. Two or three eyes, therefore, are kept at hand, each having an iris corresponding with a particular strength of light. Such are the niceties to which some wearers are said to carry the thing, while at the other end of the scale are said to be some who cannot afford to buy an eye at all; and who, if they must have one, are accustomed to hire it by the month!

